# Testing landmark-specific effects on route navigation in an ecologically valid setting: a simulated driving study

**DOI:** 10.1186/s41235-022-00374-w

**Published:** 2022-03-07

**Authors:** Yasaman Jabbari, Darren M. Kenney, Martin von Mohrenschildt, Judith M. Shedden

**Affiliations:** 1 Department of Psychology, Neuroscience and Behaviour, McMaster University, Hamilton, ON Canada; 2 Department of Computing and Software, McMaster University, Hamilton, ON Canada

**Keywords:** Spatial navigation, Proximal landmarks, Distal landmarks, Driving simulator, Virtual environment, Survey knowledge, Route learning, Wayfinding

## Abstract

We used a driving simulator to investigate landmark-based route navigation in young adults. Previous research has examined how proximal and distal landmarks influence route navigation, however, these effects have not been extensively tested in ecologically-relevant settings. We used a virtual town in which participants learned various routes while simultaneously driving. We first examined the effect of four different landmark conditions on navigation performance, such that each driver experienced one of four versions of the town with either proximal landmarks only, distal landmarks only, both proximal and distal landmarks, or no landmarks. Drivers were given real-time navigation directions along a route to a target destination, and were then tested on their ability to navigate to the same destination without directions. We found that the presence of proximal landmarks significantly improved route navigation. We then examined the effect of prior exposure to proximal vs. distal landmarks by testing the same drivers in the same environment they previously encountered, but with the landmarks removed. In this case, we found that prior exposure to distal landmarks significantly improved route navigation. The present results are in line with existing research on route navigation and landmarks, suggesting that these findings can be extended to ecologically-relevant settings.

## Significance statement

During wayfinding and navigation, landmarks influence our spatial encoding and representation of the environment. Real world navigation is often paired with complex body coordination tasks such as walking or driving rather than simple keyboard or joystick movements. In this study, we assessed whether existing route-based navigation findings extend to ecologically-relevant settings by combining navigation and driving in an immersive driving simulator. Our results suggest that guided navigation while driving (e.g., GPS systems) may be facilitated differentially by proximal vs. distal landmarks. Route navigation was facilitated when the initial guided navigation was in the presence of proximal landmarks, suggesting proximal landmarks may be more specifically associated with navigation decisions. When tested in an environment without any landmarks, prior exposure to distal landmarks in that same environment improved navigation, suggesting that distal landmarks may facilitate orientation knowledge of the space. This work may be important for designing GPS navigation systems that improve navigation by taking advantage of spatial memory.

## Introduction

As we move through a spatial environment, information about our relative position is updated. Humans are flexible navigators, actively changing navigation strategies depending on the available information such as external landmark cues in the environment (Caduff & Timpf, 2008; De Condappa, 2016; Foo et al., 2005; Siegel & White, 1975; for a review, see Montello, 2005). Landmarks can be categorized as either proximal or distal (Caduff & Timpf, 2008; Chan et al., 2012; Cohen & Schuepfer, 1980; Jansen-Osmann, 2002; Stankiewicz & Kalia, 2007; Steck & Mallot, 2000). Proximal landmarks are visible from a short distance and can provide local positional information (Chan et al., 2012; Steck & Mallot, 2000; Wilson & Alexander, 2008), whereas distal landmarks are visible from far distances (e.g., the moon or a city tower) and can facilitate acquisition of map-like orientation information (Hamilton et al., 2002; Jacobs et al., 1997; Steck & Mallot, 2000).

Landmarks are important for the generation and application of two qualitatively different types of spatial knowledge: survey and route knowledge (Evans et al., 1984; Jansen-Osmann & Fuchs, 2006; Montello, 2005; O’Laughlin & Brubaker, 1998; Waller & Lippa, 2007; Wiener et al., 2009). Survey knowledge is based on cognitive maps, which are mental representations of the environment that reflect the spatial configuration of landmarks relative to one another (O’Keefe & Nadel, 1978; Taylor et al., 1999; Thorndyke & Hayes-Roth, 1982; Tolman, 1948; Zimmer, 2004). Some work has shown that distal landmarks best support survey knowledge because they provide global orientation information about the environment (De Condappa, 2016; Hamilton et al., 2002; Jacobs et al., 1997; Livingstone-Lee et al., 2011; Mueller et al., 2008).

In contrast, route knowledge is a relatively basic navigation strategy that requires knowledge of a particular sequence of landmarks and turn decisions (Andersen et al., 2012; Daniel et al., 2003; Hartley et al., 2003; Klatzky, 1998; Siegel & White, 1975; Waller & Lippa, 2007). Proximal landmarks support route knowledge by providing intermediate goals along the route (Hurlebaus et al., 2008; Ruddle et al., 2011; Steck & Mallot, 2000). Successful navigation using route knowledge does not require survey knowledge, and tends to be less cognitively demanding but also less flexible than survey knowledge due to reliance on the *sequence* of decisions (e.g. Chan et al., 2012; De Condappa, 2016; Hartley et al., 2003; Knierim & Hamilton, 2011; Steck & Mallot, 2000; Wilson & Alexander, 2008). Moreover, the utility of proximal landmarks is limited to a local spatial area, and the view of that space changes with the viewpoint. Accordingly, using proximal landmarks to obtain allocentric information for locating a target is challenging (Benhamou & Poucet, 1998; Knierim & Hamilton, 2011; O’Keefe & Nadel, 1978; Save & Poucet, 2000).

Many navigation studies assessing route navigation used basic paradigms in which cognitive load was limited to the navigation task itself (Andersen et al., 2012; Bakdash et al., 2008; Carassa et al., 2002; Gardony et al., 2011; Hurlebaus et al., 2008; Janzen & Turennout, 2004; Knorr et al., 2014; Péruch et al., 1995; Stankiewicz & Kalia, 2007; Viaud-Delmon & Warusfel, 2014; Wallet et al., 2011). These studies were tightly controlled and provided internal validity, but we seek to extrapolate the findings to navigation in the real world. Studies that require active driving or walking while navigating in a real environment inevitably involve various confounding actions, but include sensory cues that are not available in a more limited environment, such as cues to production and detection of self-motion (Whishaw & Wallace, 2003; Poucet & Save, 2017; Lin et al., 2016; for a review see Chrastil & Warren, 2012). Moreover, actively driving and navigating associates actions with the relative spatial relations that link landmarks, routes, and allocentric directions (Appleyard, 1970; Chrastil & Warren, 2012; Duncan, 1984; Maguire et al., 2000, 2006). An immersive virtual environment can provide a more controlled and stable experimental design while bridging behaviour in the laboratory with behaviour in the real world (Appleyard, 1970; Chrastil & Warren, 2012; Farrell et al., 2003).

A more realistic driving scenario that incorporates simultaneous navigation and driving may present a more demanding situation for drivers, and this may interact with navigation strategies that are cognitively demanding, such as the use of distal landmarks for allocentric navigation (De Condappa, 2016; Waller & Lippa, 2007). Drivers may tend to be reliant on global positioning systems (GPS) for their navigation, and it has been recognized that outsourcing spatial cognition processing (Clark, 2017; Foglia, & Wilson, 2013; Gračanin, 2018; Hollan et al., 2000; Soler et al., 2017; Wilson, 2002) may result in significantly poorer spatial memory for locations (Hejtmánek et al., 2018; Ishikawa et al., 2008; Leshed et al., 2008; Münzer et al., 2006; Willis et al., 2009). Therefore, understanding how landmarks influence real-time route navigation in drivers is an important first step to understanding how the existing navigation literature extends to route navigation in the real world.

In the present study we used a driving simulator to simulate a realistic navigation environment. The first goal of our study was to examine the effect of proximal and distal landmarks on route navigation. The second goal was to examine the contribution of prior exposure to proximal and distal landmarks by assessing route navigation in that same environment without landmarks. Our hypotheses were that (1) route navigation would be sensitive to type of landmark, and that (2) route navigation in the absence of landmarks would be sensitive to prior experience with different types of landmarks in that same environment.

## Methods

### Participants

We recruited 128 participants (55 females) between the ages of 18 to 31 years old (M = 19.21, SD = 2.48). These were graduate students who participated as volunteers and undergraduate students who participated for course credit. Participants were screened based on self-reports to have no vision or hearing problems and no sensitivity to cyber sickness or claustrophobia. Self-report questionnaires also estimated familiarity with computers and computerized and virtual games. Participants were randomly assigned to one of four groups (32 per group). Six participants were removed due to technical issues. Each group received a different type of landmark during the training block: combined (proximal and distal) (PD, *n* = 30), distal (D, *n* = 31), proximal (P, *n* = 30), or no landmarks (N, *n* = 31). The experiment was approved by the Hamilton Health Research Ethics Board and complied with the Canadian Tri-Council policy on ethics.

### Experimental setup

**Fig. 1:**
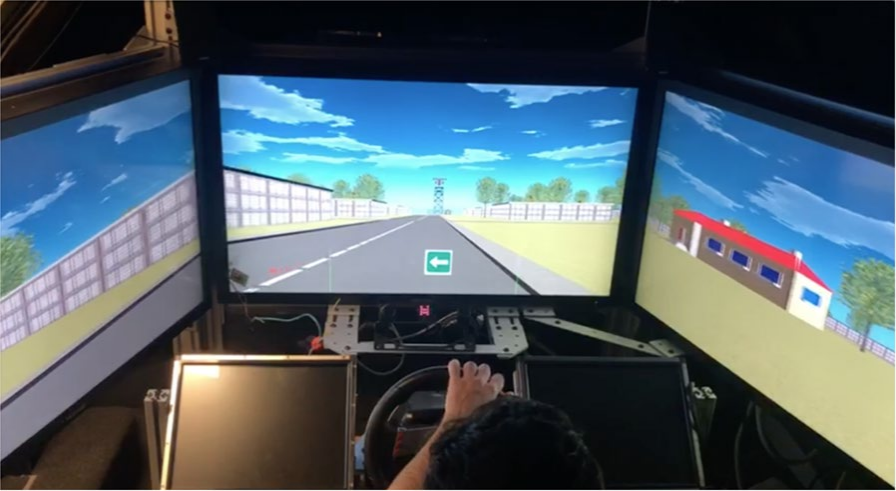
Driving simulator from the participant’s view as they completed the guided navigation phase of the combined landmark group (PD group). Participants controlled their vehicle with a steering wheel, gas, and brake pedals. The small screens on the left and right of the steering wheel were not used in this experiment. In this example, a tall radar tower at the edge of the town (distal landmark), and a colorful house at the upcoming intersection (proximal landmark) are visible to the driver. A white/green navigation arrow briefly presented at the bottom of the central screen indicates a left turn at the next intersection

Visual stimuli were presented on three 42″ (diagonal) LCD panels, each with a resolution of 1920 × 1080 pixels, running at a 60 Hz refresh rate. The LCD panels were set up in an enclosed environment and aligned in an arc to provide a wide field of view (FOV) of 35° vertically and 120° horizontally (Fig. 1). A wide FOV provides a higher viewer capacity that is more akin to driving in a real environment by increasing perception of the self-motion cues in the periphery as participants move through space and make head turn movements (e.g., Mizell et al., 2002; Narayan et al., 2005; Pausch et al., 1997; Starkweather, 2003). Research has shown that using wide FOV displays is beneficial for navigation performance (e.g., Patrick et al., 2000; Tan et al., 2006; Tyndiuk et al., 2007).

Participants sat in a bucket car seat positioned to maintain an approximate distance of 120 cm between the participants’ eyes and the central LCD screen. The driving interface consisted of a Logitech steering wheel and gas and brake pedals (Logitech International S.A., Lausanne, Switzerland). There were two cameras inside the simulator pod for experimenter monitoring purposes; one provided a front view of the participant, and the other provided a bird’s eye view of the interior. An intercom system allowed communication between the participant and the experimenter. Data from the steering wheel and pedals were continuously recorded at a sampling rate of 60 Hz.

### Virtual environment

**Fig. 2:**
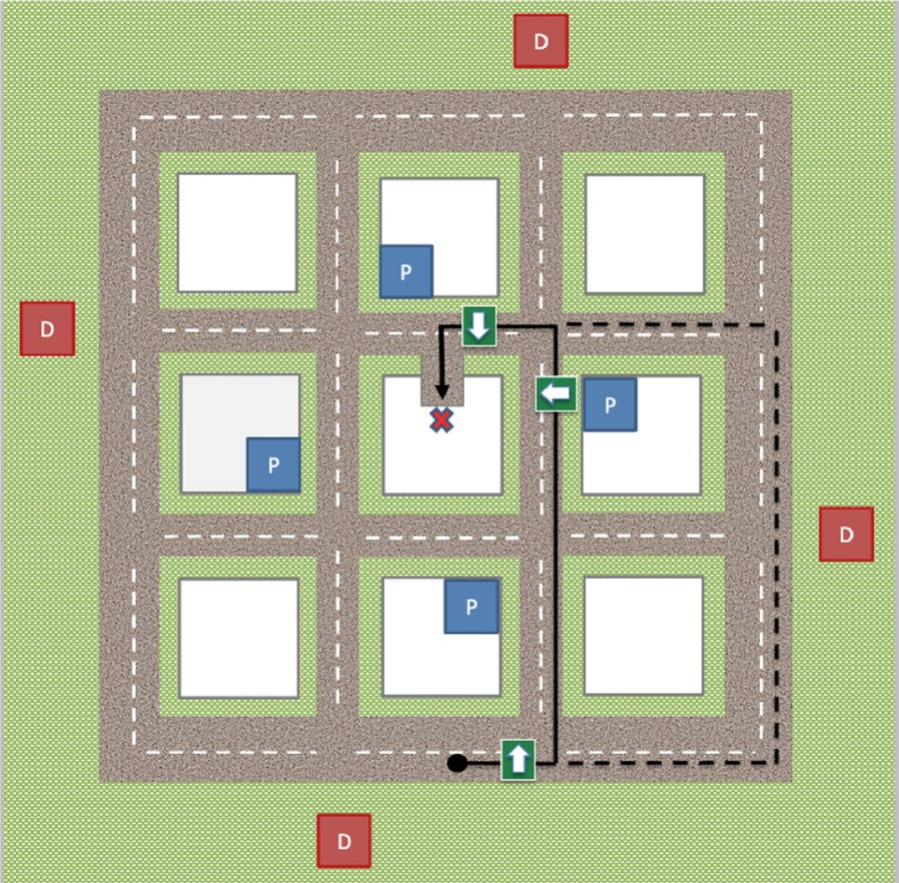
Bird’s eye view of the virtual town in the same condition (not shown to the participant). The red “D” squares indicate locations of distal landmarks, and the blue “P” squares indicate locations of proximal landmarks. An example training route is denoted with a black solid line from start position (black circle) to target location (red X). The arrows indicate the approximate point at which the white/green navigation arrows appear as the driver approaches the intersection during the guided navigation phase. The dashed black line is an example of an alternate route the driver might take during the testing phase if they failed to follow the trained route (learned in the guided navigation phase) but successfully reached the target location (e.g., partial route retracing)

The dynamic visual simulation of the town was coded in C++ using the Vega Prime (Presagis) library*.* The small virtual town consisted of a 3 × 3 grid of city blocks defined by a 4 × 4 grid of roads (Fig. 2). The target destination was a dead-end at the center of the town. All conditions included visually-similar generic buildings throughout the town (see Fig. 1), except for the landmarks which were distinctive. There were two types of landmarks: 4 proximal landmarks and 4 distal landmarks. Proximal landmarks were 3D renderings of a church, a store, a house, and a gas station. Proximal landmarks were located on corner lots at central intersections and were visually obstructed by the surrounding generic buildings so that they were only visible from nearby locations and could not be used as distal landmarks.

Distal landmarks were 3D renderings of a radar tower, a control tower, a water tower, and a wind tower, located beyond the outer boundaries of the town and tall enough to be visible from most locations in the town when facing in the right direction. In experimental conditions in which the proximal and distal landmarks were not present, the visually-similar generic buildings and the surrounding roads looked similar from each of the internal intersections of the town. Without the distal landmarks, the town’s edges were not differentiable from each other and due to the render distance were visible to navigators only when they were driving on a road close to the edge. Although there is an illusion of simplicity when looking at the top-down view of the town in Fig. 2 (top-down view was not provided to participants), navigation was challenging enough to elicit strong individual variation in route-navigation performance.

### Procedure

A demographic questionnaire collected self-report data including gender and average daily hours of driving, playing video/computerized games, and computer use outside of games. Participants practiced driving in the simulator (e.g. making left and right turns, accelerating and braking) until they were comfortable with the operation of the steering wheel, pedals, and immersive visual environment.

The experimental design consisted of 2 blocks (a Training block followed by a Transfer block), 2 phases per block (a Guided Navigation phase followed by a Testing phase), and 4 navigation trials per phase. Participants were randomly assigned to 1 of 4 groups defined by the type of landmarks presented in the Training block: both proximal and distal landmarks combined (PD), proximal landmarks only (P), distal landmarks only (D), or no landmarks (N). Importantly, landmarks were present during the Guided Navigation and Testing phases of the Training block only.

*Training block*: In the Guided Navigation phase of the Training block, participants were guided along 4 different routes. Each route had a different starting point, traversed four intersections (4 decision points), and terminated at the target destination (dead-end road in the centre of town). Navigation at each intersection was guided by a left, right, or forward arrow displayed at the bottom of the screen as the car approached the intersection. In the Testing phase, the same 4 routes were tested in the same order, but this time without the guided-navigation arrows. For each route, the car was placed at the same starting point as the guided navigation phase. Participants were instructed to drive to the target destination by retracing the learned route exactly. However, if they took a wrong turn or were unable to remember the exact route, they should try to reach the target destination via an alternate route. There was a 90 s time limit after which the trial terminated even if the participant failed to reach the target destination.

*Transfer block*: The procedure in the Guided Navigation and Testing phases was the same as in the Training block, except for two differences: (1) there were no landmarks present for any of the 4 landmark groups, and (2) a different set of 4 routes were used for all 4 landmark groups.

The Training and Transfer blocks were each about 10 min in duration and participants were offered a short break (2–4 min) after the Training block. The entire experiment was about 45 min in duration, including informed consent, the demographic questionnaire, driving practice, instructions, the two experimental blocks, and final debriefing which clarified the design and purpose of the experiment.

### Data analysis

We assessed route navigation performance in the testing phase of each Training and Transfer block by evaluating two dependent variables: success and route retracing.

*Success:* Success was the percentage of trials for which the target destination was found before the 90 s time limit. There were 4 trials (routes) in each testing phase, and the outcome of each trial was either a success or fail, thus success rate was treated as a binomial variable.

*Route retracing:* In the testing phase, participants were instructed to find the target destination by retracing the corresponding routes from the guided navigation phase, however they were permitted to take alternate routes. Route retracing included all success and failure trials to capture memory for the learned route. The route retracing score was calculated as a percentage of the length of the route taken in the testing phase that overlapped with the route taken in the guided navigation phase (see Fig. 2). A route retracing score of 100% would indicate that the route taken in the testing phase overlapped the entire length of the guided navigation route.

*Generalized linear mixed model (GLMM):* Our models included two independent fixed variables of Landmark Group (PD, P, D, & N) and Block (training & transfer). The Trial Order variable was analyzed as both fixed (Trial Order) and random (Trial Order x participants). Treating Trial Order as a fixed effect accounted for overall learning effects; treating Trial Order as a random slope across participants accounted for individual differences in learning rates. Since the dependent variable success (S) was a binomial variable (success or failure), we used a logit link function to create a logistic regression model, by applying a generalized linear mixed model (GLMM) (Agresti, 2015; Dunteman & Ho, 2006). In the regression summary, the first category is used as a reference category. Which category is selected as the reference does not affect the results, it only determines the specific comparisons that the model reports. We selected PD (training block) as the reference category. We hypothesized that due to the availability of both types of landmarks, this group would have the highest level of predicted value (success). Thus, using PD as the reference category against which the other landmark conditions are compared is an intuitive way to interpret the output of the model.

The four questionnaire variables were gender, average daily gaming computer hours, average daily non-gaming computer hours, and average daily driving hours. Of the questionnaire variables, only computer hours predicted success (*z* = 2.227, *p* = 0.026), and none predicted route retracing. Final models excluded these non-significant questionnaire predictor variables (see Wilkinson notation below). The estimate (*β*) of each term in the logistic regression model is the log-odds ratio between the term and the reference category. The odds ratio (OR) is equal to exp (*β*) and describes the ratio of the likelihood of success between the two categories. Since route retracing was a continuous variable, it was modelled with a linear mixed model (LMM) with the same independent variables. In Wilkinson notation (Wilkinson & Rogers, 1973) the models are as follows:

*Success (S):*

$${\text{S}}\,\sim \,{\text{landmark}}\,{\text{group}}\,*\,{\text{block}}\, + \,{\text{trial}}\, + \,{\text{computer}}\,{\text{hrs}}\, + \,(0\, + \,{\text{trial}}\,|\,{\text{participants}})$$

*Success learning effect:*

$${\text{S}}\sim {\text{landmark}}\,{\text{group}}\,*\,{\text{trial}} + {\text{computer}}\,{\text{hrs}} + \left( {0 + {\text{trial}}\,|\,{\text{ participants}}} \right)$$

*Route Retracing (RR):*

$${\text{RR}}\sim {\text{landmark}}\,{\text{group}}\,*\,{\text{block}} + {\text{trial}} + \left( {{1} + {\text{trial}}\,|\,{\text{participants}}} \right)$$

*Route retracing learning effect:*

$${\text{RR}}\sim {\text{landmark}}\,{\text{group}}\,*\,{\text{trial}} + \left( {{1} + {\text{trial}}\,|\,{\text{participants}}} \right)$$

## Results

### Success

**Fig. 3:**
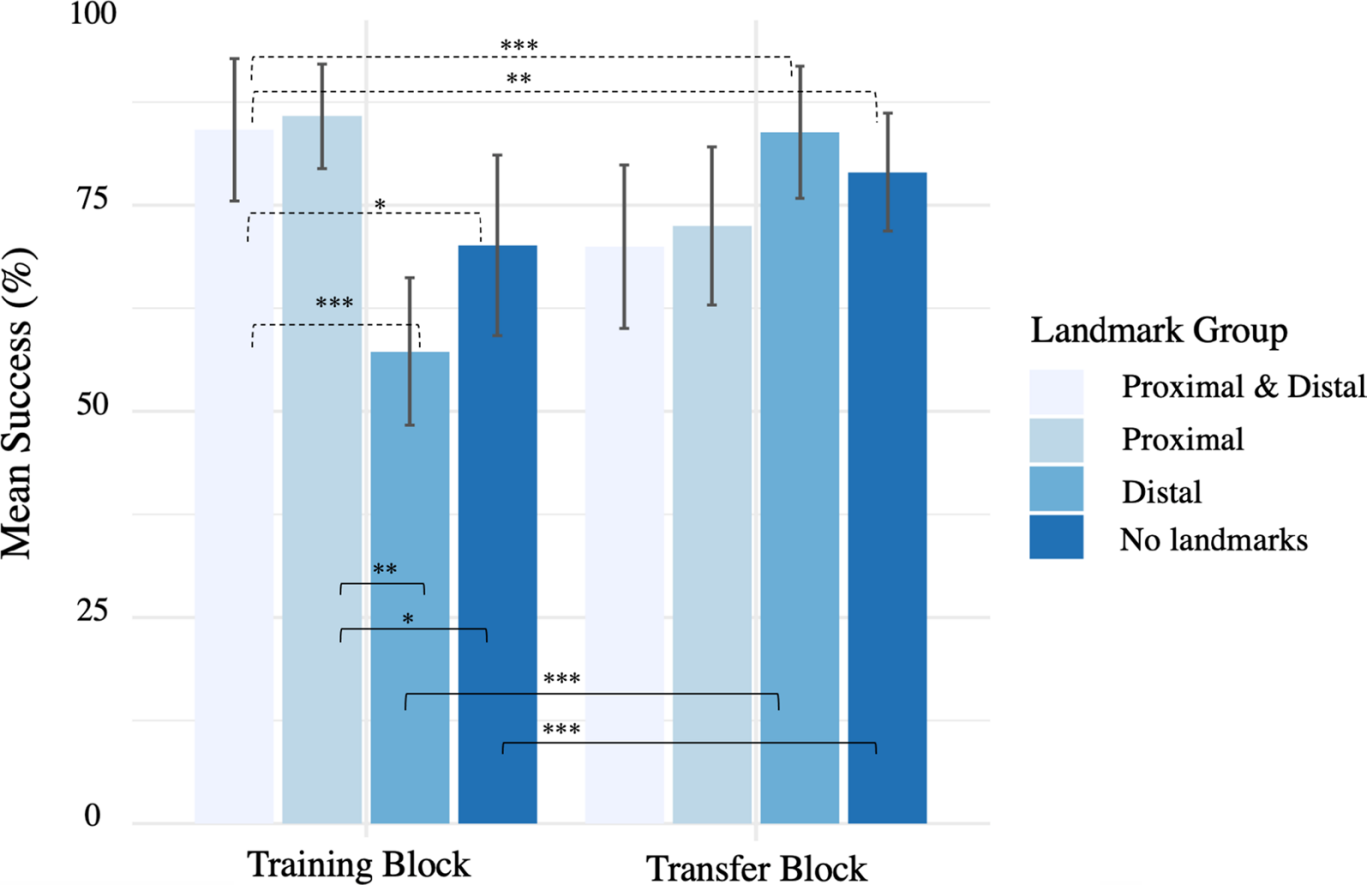
Success for each Landmark group and Block. Error bars are 95% confidence intervals. Black dashed lines indicate significant model interactions and black solid lines indicate significant pairwise comparisons. Asterisks “***”, “**”, and “*” denote *p* < .001, *p* < .01, *p* < .05, respectively

The statistical results of the generalized linear mixed model are reported in Table 1. In the training block, both the D group (*β* = − 1.370, *z* = − 4.117, *p* < 0.001) and the N group (*β* = − 0.838, *z* = − 2.495; *p* = 0.0125) were less successful in finding the target location compared to the PD group. Success of the P group did not differ from the PD group (*p* = 0.714). Significant interactions revealed that the success in PD across training and transfer blocks differed from those of D (*β* = 2.413, *z* = 4.843, *p* < 0.001) and from N (*β* = 1.490, *z* = 3.125, *p* = 0.0017). No significant interaction was observed for the P group (*p* = 0.799) (Fig. 3 and Table 1).

**Table 1:** Results of the generalized linear mixed model with success modelled by Landmark group and Block

Landmark*Block	Estimate (*β*)	SE	OR	CI (95%)		*z* value	Pr( >|*z*|)
*PD, Training Block (reference)*	2.805	0.414	16.52	2.01	3.64	6.770	< .001***
*P, Training Block*	0.138	0.378	1.15	− 0.60	0.89	0.366	0.714
*D, Training Block*	− 1.370	0.332	0.25	− 2.04	− 0.73	− 4.117	< .001***
*N, Training Block*	− 0.838	0.336	0.43	− 1.51	− 0.19	− 2.495	0.0125*
*Transfer Block*	0.254	0.443	1.29	− 0.62	1.12	0.573	0.566
*Trial Order*	− 0.261	0.074	0.77	− 0.41	− 0.12	− 3.51	< .001***
*P: Transfer Block*	0.127	0.498	1.14	− 0.85	1.11	0.254	0.799
*D:Transfer Block*	2.413	0.498	11.17	1.46	3.42	4.843	< .001***
*N:Transfer Block*	1.490	0.477	4.43	0.57	2.44	3.125	0.0017**

Asterisks “***”, “**”, and “*” denote *p* < .001, *p* < .01, *p* < .05, respectively

Pairwise comparisons with Tukey adjustments were used to test the difference in success between the training and transfer block of each group (a repeated-measures comparison). Success in the D group was significantly lower in the training block compared with the transfer block (Training: M = 0.572, SE = 0.044 vs Transfer: M = 0.838, SE = 0.033; *β* = − 2.667, *z* = − 5.656, *p* ≤ 0.0001). Similarly, success in the N group was lower in the training block (Training: M = 0.701, SE = 0.041 vs Transfer: M = 0.790, SE = 0.036; *β* = − 1.744, *z* = − 3.938,* p* ≤ 0.0001). No significant difference was observed between the training and transfer block for the P group (Training: M = 0.858, SE = 0.031 vs Transfer: M = 0.725, SE = 0.040; *β* = − 0.380, *z* = − 0.830, *p* = 0.407) or for the PD group (Training: M = 0.841, SE = 0.033 vs Transfer: M = 0.700, SE = 0.042; *β* = − 0.254,* z* = − 0.573, *p* = 0.567). Additional pairwise comparisons assessing the success difference between groups within each block showed that, in the training block, success of the P group was significantly higher than the D group (*β* = 1.509, *z* = 4.399, *p* = 0.001) and the N group (*β* = 0.977,* z* = 2.823, *p* = 0.024). There were no other significant pairwise comparisons between the non-referenced groups. Overall, these results suggested that prior exposure to distal landmarks or no landmarks may have facilitated later route navigation in the same environment when no landmarks were present.

### Success learning effect

There was a significant effect of Trial Order on success (*β* = − 0.261, *z* = − 3.51, *p* < 0.001), such that success improved over time. We analyzed interactions between Landmark groups and Trial Order to see if the learning effect differed across groups. We observed a significant interaction of the learning effect for success in the D group (*β* = 0.360, *z* = 3.493, *p* ≤ 0.001) as well as the N group (*β* = 0.207, *z* = 2,040, *p* = 0.041) compared to the PD group, suggesting a positive learning effect across trials for these two groups. We did not observe a significant interaction for the P group (*β* = − 0.075, *z* = − 0.683, *p* = 0.494).

### Route retracing

Results of the linear mixed model for Route Retracing are reported in Table 2. In general, route retracing scores had a strong positive correlation with success (*r* = 0.619, *p* < 0.001). Marginal mean route retracing scores (percent) of successful trials in both training and transfer blocks was high (M = 79.3, SE = 0.96) compared to failed trials (M = 31.7, SE = 1.68).

**Table 2:** Results of the linear mixed model with route retracing modelled by Landmark group (PD, P, D & N) and Block (Training & Transfer)

Block*Landmark	Estimate (*β*)	SE	CI (95%)		*t* value	Pr( >|*t*|)
*PD, Training Block (Intercept)*	82.944	4.220	74.66	91.23	19.653	< 0.001***
*P, Training Block*	9.886	4.969	0.05	19.73	1.990	0.049*
*D, Training Block*	− 8.091	4.929	− 17.85	1.67	− 1.642	0.103
*N, Training Block*	− 2.768	4.929	− 12.53	6.99	− 0.562	0.576
*Transfer Block*	14.208	5.392	3.63	24.79	2.635	0.009**
*Trial Order*	− 5.518	0.886	− 7.26	− 3.78	− 6.229	< 0.001***
*P, Transfer Block*	− 7.340	6.073	− 19.26	4.58	− 1.209	0.227
*D, Transfer Block*	16.585	6.024	4.76	28.41	2.753	0.006**
*N, Transfer Block*	11.341	6.024	− 0.48	23.16	1.883	0.060

Asterisks “***”, “**”, and “*” denote *p* < .001, *p* < .01, *p* < .05, respectively

**Fig. 4:**
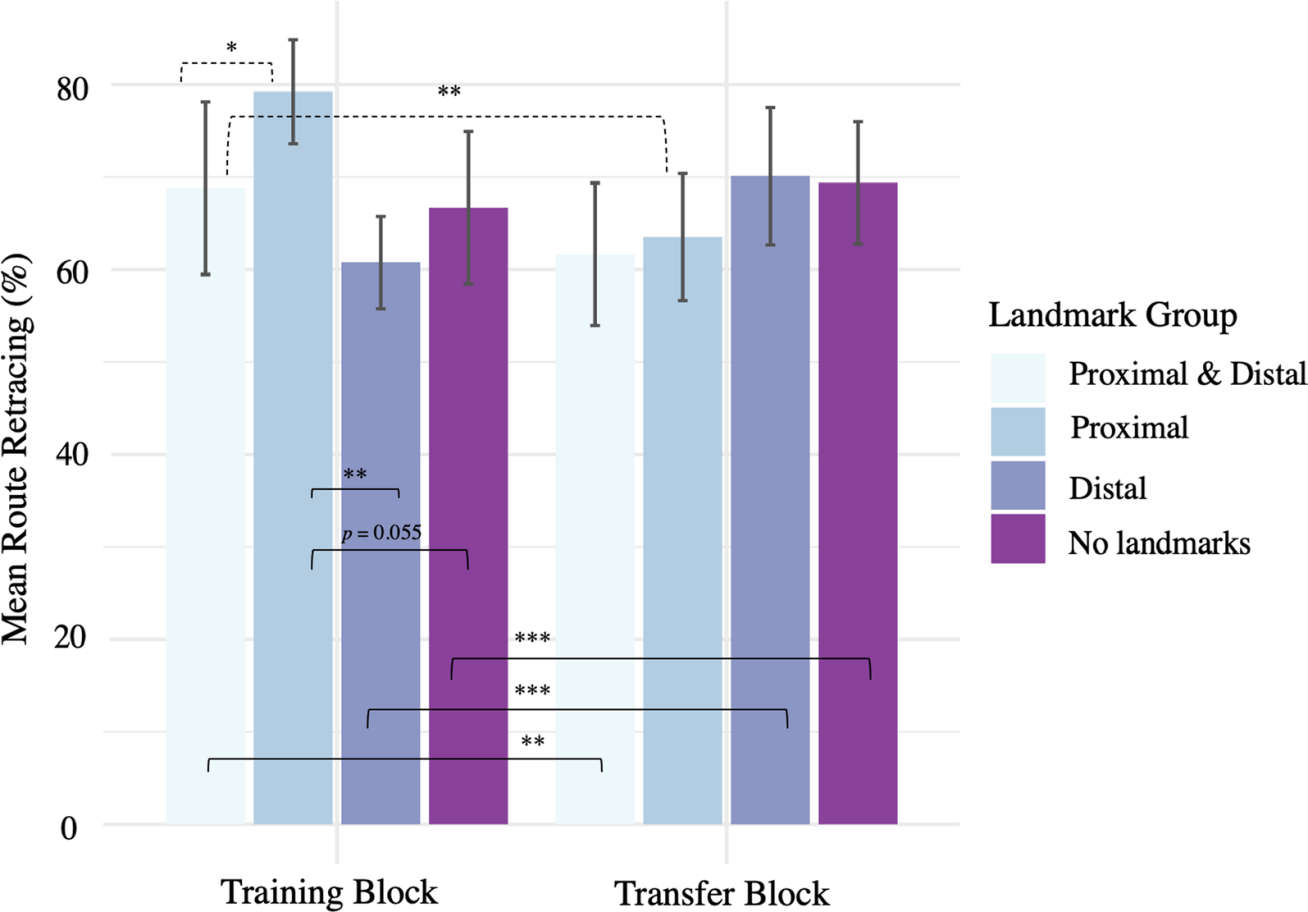
Route retracing for each Landmark group and Block, for all trials. Error bars are 95% confidence intervals. Black dashed lines indicate significant model interactions and black solid lines indicate significant pairwise comparisons. Asterisks “***”, “**”, and “*” denote *p* < .001, *p* < .01, *p* < .05, respectively

The P group had significantly higher route retracing scores than the PD group (*β* = 9.886,* t* = 1.990, *p* = 0.049). Route retracing scores in the D group (*p* = 0.103) and the N group (*p* = 0.576) did not differ from the PD group. A significant interaction revealed that the route retracing in PD across training and transfer blocks differed from those of D (*β* = 16.585, *t* = 2.753, *p* = 0.006) (Fig. 4). There was no significant interaction for the N group (*p* = 0.060) or the P group (*p* = 0.227) (Table 2).

Repeated measures pairwise comparisons showed that the route retracing scores of the D group (Training: M = 60.79, SE = 2.85 vs Transfer: M = 70.13, SE = 2.95; *β* = − 30.79, *t* = − 5.770, *p* < 0.0001), N group (Training: M = 66.73, SE = 3.07 vs Transfer: M = 69.40, SE = 3.02; *β* = − 25.55, *t* = − 4.788, *p* < 0.0001), and the PD group (Training: M = 68.84, SE = 3.12 vs Transfer: M = 61.67, SE = 3.11; *β* = 14.21, *t* = − 2.635, *p* = 0.008) were significantly different in the training block compared with the transfer block. No significant difference was observed between training and transfer blocks of the P group (Training: M = 79.25, SE = 2.66 vs Transfer: M = 63.54, SE = 3.02; *β* = − 6.87, *t* = − 1.274, *p* = 0.203). Pairwise comparison within blocks indicated that, in the training block, route retracing scores of the P group were significantly higher than the D group (*β* = 17.977, *t* = 3.647, *p* = 0.002) and marginally higher than N group (*β* = 12.654, *t* = 2.567, *p* = 0.055). No other significant pairwise comparisons were observed between the non-referenced groups. Overall, these results suggested that in the presence of proximal landmarks only (P group) drivers were more likely to retrace the exact training routes. Moreover, similar to the success results observed in the transfer block, prior driving experience in the presence of distal landmarks appeared to improve route retracing performance in the transfer block in which no landmarks were present.

### Route retracing learning effect

A significant effect of Trial Order on route retracing was observed (*β* = − 5.518, *t* = − 6.229, *p* < 0.001). There was a significant interaction of the learning effect for route retracing scores in the D group (*β* = 3.082, *t* = 2.256, *p* = 0.0243) compared to the PD group, indicating a positive learning effect across the order of trials.

## Discussion

Navigation research has shown that there are differences in the way that proximal vs. distal landmarks influence survey vs. route knowledge (Bullens et al., 2011; De Condappa, 2016; Doeller & Burgess, 2008; Hurlebaus et al., 2008; Steck & Mallot, 2000; Wilson & Alexander, 2008). Our goal in the present study was to understand whether and how these findings extend to route navigation while actively driving. We used a driving simulator to assess route navigation memory in an ecologically-relevant virtual town. We manipulated the presence of different types of landmarks in the training block and tested the effect of prior exposure to different types of landmarks on route navigation in the transfer block where no landmarks were available. In each block, we operationalized route navigation performance in two ways: “success”, which was the binomial record of the passed or failed trials in the testing phase, and “route retracing”, which was the percentage of overlap between the routes taken in the guided navigation and testing phase.

### Success

In the training block, groups exposed to proximal landmarks (PD and P groups) had significantly higher success than groups exposed to distal or no landmarks (D and N groups). This is consistent with past research showing that proximal landmarks support route knowledge (e.g., Hurlebaus et al., 2008; Ruddle et al., 2011). Proximal landmarks provide local relative positional information (Benhamou & Poucet, 1998; Biegler & Morris, 1996) which is useful in route-based navigation tasks. Even though the presence of proximal landmarks in the training block significantly improved route learning, the relatively poorer performance of the PD compared to the D group in the transfer block suggested that the mere presence of the distal landmarks is not what facilitated successful route navigation, but rather the lack of proximal landmarks. In other words, drivers' successful use of proximal landmarks when they were available resulted in less well-formed survey-like representations of the environment; this hurt performance when subsequently tested in the absence of landmarks (transfer block). We suggest that when proximal landmarks were not available in the training block (e.g. the D and N groups), drivers formed a more effective map-like representation of the environment which supported route navigation in the absence of landmarks in the transfer block.

### Route retracing

The same general pattern of results was observed in the route retracing measure, corroborating the success results. The route retracing score was an indicator of successful guided navigation phase route learning. Participants were encouraged to navigate by retracing the routes learned in the guided navigation phase, which were the most efficient routes to the target destination, but were allowed to take alternate routes.

In the training block, the P group had significantly higher route retracing scores than all other groups (PD, D, & N groups). This suggests that in the presence of proximal landmarks drivers are more likely to retrace the learned routes from the guided navigation phase, whereas in the presence of distal landmarks or absence of proximal landmarks drivers can afford more flexible navigation. Drivers benefit from proximal landmarks during navigation as they can be associated directly to turns at intersections. Distal landmarks do not provide the same highly reliable local positional information because they are too distant to link a specific turn direction to a particular intersection. However, drivers may still attend to distal landmarks when learning a route and this will lead to better map-like knowledge.

In the transfer block, where landmarks were not available, prior exposure to distal landmarks increased the retracing scores of the D group. This could mean that even though distal landmarks were not as useful to route retracing as proximal landmarks, they did help to generate a mental representation of the town which was helpful for route retracing in the transfer block. An important question is why precise route retracing in the PD group was lower when compared to the P group in the training block or D group in the transfer block. Presence of both types of landmarks together might draw attention away from efficient use of the navigational strategy that uses either type of landmarks. Efficient navigation requires the ability to dynamically weight different navigation strategies based on available landmarks (Montello, 2005; Steck & Mallot, 2000; Wiener et al., 2004, 2009). Drivers in the PD group may have attempted to use both proximal and distal landmarks, leading to a lower route retracing score.

## Summary

Many navigation studies have analyzed joystick or keyboard responses in tasks involving desktop virtual environments (e.g., Andersen et al., 2012; Bakdash et al., 2008; Carassa et al., 2002; Gardony et al., 2011; Hurlebaus et al., 2008; Janzen & Turennout, 2004; Knorr et al., 2014; Péruch et al., 1995; Stankiewicz & Kalia, 2007; Viaud-Delmon & Warusfel, 2014; Wallet et al., 2011). Some studies have used both real and virtual setups (e.g., Farrell et al., 2003; Grant & Magee, 1998), and several have examined navigation while participants walked in the real world (e.g., Münzer et al., 2006; Wallet et al., 2008; Willis et al., 2009; Zhong & Kozhevnikov, 2016). A few studies have assessed navigation and driving in the real world (e.g., Antrobus et al., 2016; Fu et al., 2019; Stülpnagel & Steffens, 2012), and only a few have tested navigation using an immersive virtual reality driving simulator (e.g., Cochran & Dickerson, 2019; Kunishige et al., 2019; Pankok Jr & Kaber, 2018; Smyth, 2007; Yi et al., 2015). It could be argued that navigating while driving is substantially more difficult than navigating by means of keyboard or joystick for multiple reasons. There is additional cognitive load due to following the rules of the road, staying in the lane, and making turns at the appropriate speed. Additional sensorimotor control involved in driving with a steering wheel and pedals might also impact navigation performance. Other studies have demonstrated that cognitive load (e.g. cell phone use) while driving negatively impacts memory for landmarks (e.g. Blalock et al., 2014). It is possible that the increased cognitive load could influence spatial memory and therefore route navigation.

We assessed navigation in an ecologically valid setup. We provided a large-scale virtual environment which required an active application of wayfinding strategies (e.g., Diersch & Wolbers, 2019; Loomis et al., 1993; Poucet, 1993; Wolbers & Wiener, 2014; Zhang et al., 2014). Size of the environmental space plays an important role in the emergence and development of allocentric survey-based navigation strategies from egocentric route-based navigation strategies (Ekstrom & Isham, 2017; Siegel & White, 1975; Török et al., 2014; Zhang et al., 2014; Zhang et al., 2014). For the D group in the present study, the development of survey knowledge was not observed immediately in the training block, but rather later in the transfer block. This is in accordance with theories indicating that the development of navigational strategies is hierarchical, with egocentric route-based strategies emerging before more cognitively demanding allocentric map-based strategies (e.g., Gagnon, et al, 2014; Han & Becker, 2014; McNamara et al., 1989; Siegel & White, 1975).

The results of the present study suggest that landmark-specific effects on route navigation in the extant literature do carry over to the elevated difficulty of navigating while driving in complex virtual environments. Specifically, we found that the presence of proximal landmarks was more likely to result in successful route navigation in the training block. In contrast, the presence of distal landmarks in the training block was more likely to result in successful route navigation in the transfer block, suggesting that drivers may have obtained survey knowledge from the distal landmarks that carried over to route navigation when no landmarks were present.

### Limitations and future approaches

In this study, we used distal and proximal landmarks that simulated those found in a typical urban driving environment. Objects that are more conspicuous relative to their environment tend to function as more salient landmarks (e.g., Caduff & Timpf, 2008; Sorrows & Hirtle, 1999). Our salient landmarks were present in the training block only and not the transfer block. A limitation of this design is that landmarks do not tend to disappear in a real environment. A future experiment might set up a scenario to explain the removal of the landmarks (e.g. major construction underway to replace infrastructure in the town). Other tests for survey knowledge could be employed. An advantage to the present design is that it retains active driving in the transfer block, which may be important in revealing transferred route knowledge.

The time required to drive from the starting point to the target destination limited the number of routes tested in each condition, which limited our ability to perform reliable statistical comparisons on route retracing for successful and failed trials separately. Future study designs should address this.

We asked participants to report their driving frequency in an average week. It would have been informative to know their overall driving experience by asking for years of driving experience, because it is conceivable that beginner drivers might experience higher cognitive load from simultaneous driving and navigation compared to more experienced drivers.

We designed the town to be relatively small with only four turns per route to avoid cybersickness. A larger town would provide additional flexibility to incorporate a larger number of routes to test different arrangements of the landmarks. No participants dropped out of the present experiment due to cybersickness, although they were given multiple opportunities to do so without penalty, however, a larger virtual environment might increase the probability of cybersickness. There are various approaches that could overcome this limitation and make it a more representative task. For example, reducing the number of stops and turns, and restricting optic flow to a smaller field-of-view has been shown to reduce cybersickness (Lin et al., 2002). The presence of vestibular motion cues may also reduce cybersickness, however this effect remains controversial (Keshavarz et al., 2018; Weech et al., 2020).

## Data Availability

The datasets, R code, examples of route retracing plots, and Omnibus test results tables are publicly available at: https://github.com/Jabbariy/Manuscript1_CRPI_2022.

## Abbreviations

PD group: Both proximal and distal landmarks
P group: Proximal landmarks
D group: Distal landmarks
N group: No landmarks
